# Administration of Spermidine and Eugenol Demonstrates Anti-Tumorigenic Efficacy on Metastatic SW620 and Primary Caco-2 Colorectal Cancer Spheroids

**DOI:** 10.3390/ijms252413362

**Published:** 2024-12-13

**Authors:** Silvia Dilloo, Anne Whittaker, Xinyue Chang, Eros D’Amen, Enzo Spisni, Silvana Hrelia, Cristina Angeloni, Marco Malaguti, Giovanni Dinelli, Francesca Truzzi

**Affiliations:** 1 Department of Agricultural and Food Sciences, Alma Mater Studiorum—University of Bologna, 40127 Bologna, Italy; silvia.dilloo2@unibo.it (S.D.); anne.whittaker@unibo.it (A.W.); xinyue.chang2@unibo.it (X.C.); eros.damen2@unibo.it (E.D.); giovanni.dinelli@unibo.it (G.D.); 2 Department of Biological, Geological, and Environmental Sciences, Alma Mater Studiorum—University of Bologna, 40127 Bologna, Italy; enzo.spisni@unibo.it; 3 Department for Life Quality Studies, Alma Mater Studiorum—University of Bologna, 47921 Rimini, Italy; silvana.hrelia@unibo.it (S.H.); cristina.angeloni@unibo.it (C.A.); marco.malaguti@unibo.it (M.M.)

**Keywords:** spermidine, eugenol, SW620, Caco-2 spheroids, co-culture intestinal equivalents, anti-tumorigenic efficacy, apoptosis, migration

## Abstract

The anti-cancer potential of eugenol (EUG) is well recognized, whereas that of spermidine (SPD) is subject to dispute and requires further research. The anti-tumorigenic potential of wheat germ SPD (150 µM) and clove EUG (100 µM), alone, in combination as SPD+EUG (50 µM + 100 µM) and, as a supplement (SUPPL; 0.6 µM SPD + 50 µM EUG), was investigated on both metastatic SW620 and primary Caco-2 colorectal cancer (CRC) spheroids. Compared to untreated controls, all treatments significantly reduced the vitality and spheroid area, increased the necrotic area, and induced apoptosis on both cell-type spheroids after 96 h, with a reduced migration evident in 2D (two-dimensional) cultures after 48 h. The comparable anti-CRC effects of the SPD+EUG and the SUPPL reflected a wide-range dose efficacy of SPD and EUG. It is of note that SPD+EUG induced a synergistic effect on the increased caspase-3 expression and reduced the migration percentage in SW620. In more physiologically relevant intestinal equivalents (healthy enterocytes [NCM460], fibroblasts [L929], and monocytes [U937]) containing embedded SW620/Caco-2 spheroids, SPD+EUG administration significantly reduced the spheroid CEA marker and proliferation, whilst simultaneously increasing occludin, autophagy LC3-II expression, and monocyte differentiation, compared to the control models. Exogenous SPD, alone and in combination with EUG, displayed an anti-CRC potential on tumor growth and metastasis, and warrants further investigation.

## 1. Introduction

Globally, CRC is the third most common cancer, and the second leading cause of tumor-related deaths, with a predominance in countries with a high to very high Human Development Index (HDI) [1,2]. Mortality by colon cancer is predicted to double by 2040 in both high and low HDI countries [1,3]. This will also include increases in early onset CRC [3,4]. CRC development is a multi-step process, and each step is associated with mutagenic and/or epigenetic events, respectively. In the classic CRC development pathway, the initiation step is triggered by mutations in the normal intestinal epithelium occurring at the *APC* gene, which is responsible for early adenoma formation [5,6]. The promotion step is then associated with cell proliferation and the activation of the *KRAS* proto-oncogene, resulting in the transformation to an intermediate adenoma. Thereafter, progression proceeds to a clinically detectable late adenoma, followed by carcinoma development, with consequent tumor invasion resulting in metastasis [5,6]. Deregulated activation of the phosphoinositide 3-kinase (PI3K) pathway by multiple genes (considered a hallmark of cancer) is involved in the proliferation, survival, migration, and invasion of numerous cancers, including CRC [7]. An involvement of bile acids in CRC has also been reported [8].

Surgery, chemotherapy, and radiotherapy are the main clinical therapies for CRC, with adenocarcinomas accounting for approximately 96% of CRCs [9]. Given the urgent need to develop therapeutic approaches that are able to combine anti-tumor effects with lower cytotoxic side effects, natural product-derived drugs have become a “hotspot” in cancer research [10,11]. Within this context, the anti-CRC mechanisms of various natural products have been the subject of several recent reviews [12,13,14,15]. Natural products can target the preclinical (initiation or promotion) stages of carcinogenesis, as well as the clinical stages (proliferation, migration, invasion). The anti-CRC mechanisms of natural products in the preclinical and therapeutic stages have been shown to include a pro-oxidant potential in promoting autophagy and apoptosis, as well as inhibitory effects on signaling/metabolic pathways involved in proliferation, migration, and invasion [12,13,14,15]. Of interest in oncological research is the use of combination drugs to promote additive or synergistic effects in the treatment response [16,17,18,19].

As one of the European Institute for Innovation & Technology (EIT) FOOD projects, a SUPPL containing clove essential oil EUG absorbed onto the cold-pressed fibrous matrix of wheat germ-containing SPD was produced [20]. The SUPPL was shown to be effective in reducing inflammatory parameters and increasing autophagy in human Caco-2 and NCM460 cell lines with no cytotoxic side effects [20]. In conjunction with a healthy diet, the SUPPL was recommended for individuals with chronic intestinal inflammation [20], according to its potential as an anti-inflammatory preventative strategy against CRC. Furthermore, from the same research group, the essential oil EUG was shown to induce apoptosis, necrosis, and cell cycle retardation in the cancer cell lines of Caco-2 and SW620 but not in the control NCM460 cell lines [21].

Recent reviews have highlighted the undisputed anti-cancer potential of EUG using several cancer cell lines [22,23,24]. Specifically focusing on various CRC cell lines, EUG was shown to promote apoptosis, tumor suppression genes, and cell cycle arrest in 2D cultures [25,26,27]. For SPD, epidemiological studies demonstrated that increased nutritional uptake of SPD is linked to reduced CRC risk and mortality [11,28,29]. Despite the reduced CRC risk attributed to SPD, SPD has ironically been implicated in the growth of solid tumors. During tumorigenesis, SPD/spermine N1-acetyltransferase (SSAT; a biomarker of several cancers) increases, resulting in increased intracellular SPD production, as well as increased cancer growth, including that of CRC [19,30,31]. Polyamine analogues that reduce polyamine pools, in combination with therapeutic drugs, were reported to induce cell death [30,31]. In July 2023, a model was put forward, hypothesizing that in the tumor microenvironment, endogenous production of SPD promotes cancer proliferation and survival, but that exogenous SPD supplementation elicits anti-tumor responses. These anti-tumor responses are suggested to act by targeting pathways that improve immune cell function and override the tumor-promoting functions [31]. The promising anti-cancer potential of SPD was also highlighted in recent reviews [19,30].

There is a requisite for future studies on the anti-tumorigenic effect of SPD supplementation on different tumor types, as the available literature is scarce [19,31]. In contrast, the anti-CRC mechanisms of EUG have been individuated. However, to the best of our knowledge, all previous experiments were conducted on cell lines in 2D [19,24,25], and not on 3D (three-dimensional) cancer spheroids, as in vitro models for solid tumors. Presently, it is widely reported that spheroids offer the possibility to recapitulate the structural and molecular complexity of cancer cell lesions with higher physiological relevance compared to 2D culture systems. Hence, spheroids and other 3D in vitro models are more suitable for evaluating therapeutic responses in CRC drug research. Since there is an interest in both natural drugs and drug combinations in oncological research [12,13,14,15,16,17,18,19], the present study was focused on examining the anti-CRC potential of exogenous SPD supplementation, combined with essential oil EUG. To date, this combination has not yet been examined. Thus, the aim of this study was to investigate the anti-tumorigenic effects of SPD and EUG individually, as well as in combination as SPD+EUG, and as a supplement. SPD and EUG were investigated on the SW620 cell line (human colon cancer cells derived from lymph node metastatic site, high-grade tumor) in the 3D homotypic spheroid form. Comparisons were also made to the effects on the more extensively used Caco-2 cell line (human colon primary epithelial adenocarcinoma, low-grade tumor) in the 3D homotypic spheroid form. Moreover, anti-CRC efficacy was also examined in physiologically more relevant reconstructed healthy intestinal mucosa models containing embedded SW620/Caco-2 spheroids.

## 2. Results

### 2.1. Effects of SPD and EUG Alone and in Combination on the Proliferation, Structure, and Viability of Metastatic SW620 Spheroids Compared to Untreated Controls

**Figure 1:**
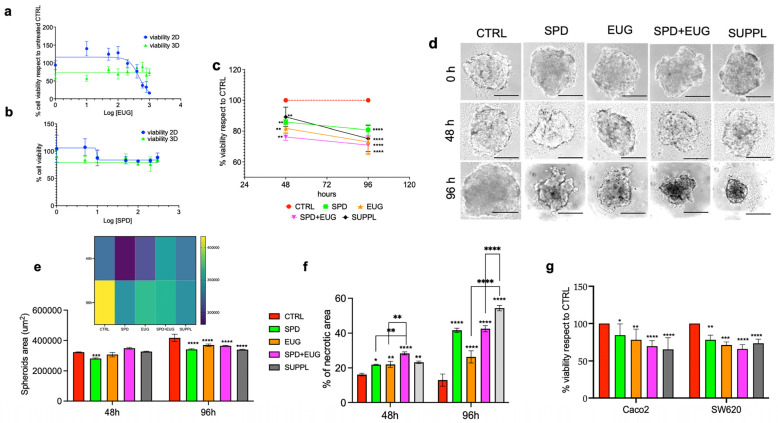
Viability of SW620 spheroids (green line) and SW620 cells cultured in 2D (blue line) after 24 h treatment with (**a**) EUG (1–1000 µM) and (**b**) SPD (1–300 µM). SW620 spheroid (**c**) viability, (**d**) morphology, (**e**) area and (**f**) necrotic area following 48 h- and 96 h-exposure to SPD (150 µM), EUG (100 µM), SPD+EUG (150 µM SPD + 100 µM EUG) and the SUPPL (0.6 µM SPD + 50 µM EUG). SW620 and Caco-2 spheroid viability (**g**) after a 96 h-exposure to the treatments. All treatments were compared to the untreated CTRL. The MTT assay (**a**–**c**) for all treatments was performed and the data reported as a percentage of the CTRL. From the light microscopy images of the spheroids at a magnification of ×20 (**d**), the areas and necrotic zones were calculated (**e**,**f**) with the area also expressed using a color heatmap to better visualize the differences. The scale bar is 50 µm. Significant differences were reported as follows: * *p* < 0.05, ** *p* < 0.01, *** *p* < 0.001, **** *p* < 0.0001.

Given that cancer cells have been shown to exhibit a greater sensitivity to PI3K inhibitors when cultured in 3D compared to 2D [16,32,33,34], and given that EUG is a PI3K inhibitor [27,35], it was considered important to first compare the dose-dependency of EUG on cells in both 2D and 3D (spheroid) forms. EUG concentrations ranging from 1–1000 µM were administered to SW620 cells in both 2D and 3D for 24 h (Figure 1a). Viability was measured using the 3-(4,5-dimetiltiazol-2-il)-2,5-difeniltetrazolium (MTT) assay. There was no dose-dependent response between 1–1000 µM EUG. The viability percentage remained constant, at approximately 75%. SW620 spheroids showed significantly greater sensitivity to EUG between 1–200 µM than cells that were cultured in 2D. The viability in 2D cells was approximately 115% of the untreated CTRL in the range of 1–100 µM EUG. Thereafter, EUG became toxic, and the viability decreased to below 50% between 600–1000 µM (Figure 1a). Similarly, the dose-dependency of SPD (1–300 µM) was examined on SW620 cells in both 2D and 3D (Figure 1b). There was no dose-dependent response between 1–300 µM SPD in the 3D spheroids. Between 1–5 µM SPD, viability was significantly more affected in 3D (80–90%) compared to 2D (110%). Thereafter, the viability between 10–300 µM was equivalent for both 3D and 2D (Figure 1b).

Since the level of sensitivity to both EUG and SPD in spheroids was shown to span a broad concentration range, subsequent experiments were performed at a selected SPD and EUG concentration. The concentrations were 150 µM and 100 µM for SPD and EUG, respectively. SPD and EUG were administered both alone and in combination. The first combined treatment was performed by adding concentrations reflecting those of the individual components alone, which are as follows: SPD (150 µM) + EUG (100 µM). In the second combined treatment, the powdered SUPPL wheat germ matrix was diluted to generate significantly lower concentrations of 0.6 µM SPD, with a corresponding absorbed EUG content of 50 µM. For each parameter investigated, an untreated control (CTRL) lacking any treatment was included.

The efficacy of the treatments on in vitro models for solid tumors would necessitate a reduction in the proliferative capacity and spheroid size. The SW620 spheroids were allowed to develop for a period of 72 h, prior to the administration of the different treatments. The anti-proliferative efficacy was then examined using the MTT assay after 48 and 96 h, respectively, and expressed as a percentage of the CTRL (Figure 1c). All treatments induced a significant reduction in SW620 spheroid viability after 48 h compared to the CTRL. A further significant decline in viability was observed between 48 and 96 h with EUG alone, SPD+EUG, and the SUPPL (Figure 1c).

The SW620 spheroids were then visualized under light microscopy at time zero, which was the moment before adding the treatments. Spheroids were also shown 48 h and 96 h after treatment exposure. A cell number of 500 was selected, as higher cell numbers were shown to produce spheroids that were too compacted over time. The untreated CTRL spheroids increased from time zero to 96 h (Figure 1d). The spheroid areas (Figure 1e) and internal necrotic areas (Figure 1f) were then quantified for each treatment. At 96 h, the spheroid area of the CTRL was significantly larger than the spheroid areas exposed to all treatment combinations (Figure 1e). The necrotic areas were higher in all treatment combinations compared to the CTRL after 48 h. The necrotic areas were shown to continue to increase significantly after 96 h (Figure 1f). At 96 h, the necrotic areas were significantly higher in the combination treatments compared to the individual treatments. This was particularly evident for the SUPPL treatment (Figure 1f).

Vitality (Trypan Blue) was investigated in both the SW620 and Caco-2 spheroids after 96 h (Figure 1g). Vitality was significantly reduced after all treatments in both cell types compared to the untreated CTRL. Relative to the untreated CTRL, the combination treatments reduced vitality to a comparable degree in both cell types (Figure 1g).

### 2.2. Effects of SPD and EUG Alone and in Combination on the Proliferation and Structure of Primary Caco-2 Spheroids Compared to the Untreated CTRLs

**Figure 2:**
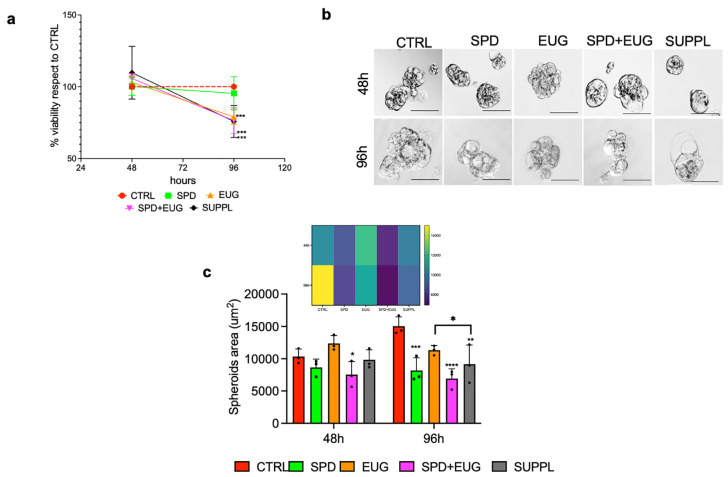
Caco-2 spheroid (**a**) viability, (**b**) morphology and (**c**) area following 48 h- and 96 h-exposure to SPD and EUG treatments as follows: SPD (150 µM), EUG (100 µM), SPD+EUG (150 µM + 100 µM) and the SUPPL (0.6 µM SPD + 50 µM EUG). All treatments were compared to the untreated CTRL. (**a**) The MTT assay for all treatments was performed and the data reported as a percentage of the CTRL. From the light microscopy images of the spheroids at a magnification of ×20 (**b**), the area was calculated and also expressed using a color heatmap to better visualize the differences. The scale bar is 50 µm. Significant differences were reported as follows: * *p* < 0.05, ** *p* < 0.01, *** *p* < 0.001, **** *p* < 0.0001.

The anti-proliferative capacity of the different treatments was similarly investigated for the primary Caco-2 line. In contrast to the SW620 spheroids (in which the treatments showed an earlier efficacy, Figure 1c), there was no significant difference in the proliferative capacity of the untreated CTRL and treated Caco-2 spheroids after 48 h (Figure 2a). Between 48 h and 96 h, the CTRL spheroids were 100% viable. Spheroids treated with SPD alone were minimally affected and not significantly different from the CTRL. At 96 h, SPD+EUG, SUPPL, and EUG alone were more effective than the SPD treatment at reducing cell viability. Viability was reduced to approximately 75% of the respective CTRLs in both cell types (Figure 1c and Figure 2a).

The Caco-2 spheroids were then visualized under light microscopy after 48 h and 96 h, respectively (Figure 2b). Unlike the SW620 spheroids, which were more spherical and larger in area, the Caco-2 spheroid structure was small, loose, and bubble-shaped (Figure 2b). In the untreated CTRL, the spheroid area increased significantly between 48 and 96 h (Figure 2c). In contrast to the CTRL, the area of spheroids exposed to all treatments was significantly lower at 96 h, indicating the efficacy of the treatments between 48 and 96 h. Interestingly, compared to the untreated CTRL, SPD was shown to reduce the area of the spheroids after 96 h (Figure 2c), without having an effect on spheroid viability (Figure 2a).

### 2.3. Effects of SPD and EUG Alone and in Combination on Apoptosis in Homotypic SW620 and Caco-2 Spheroids Compared to Untreated CTRLs

**Figure 3:**
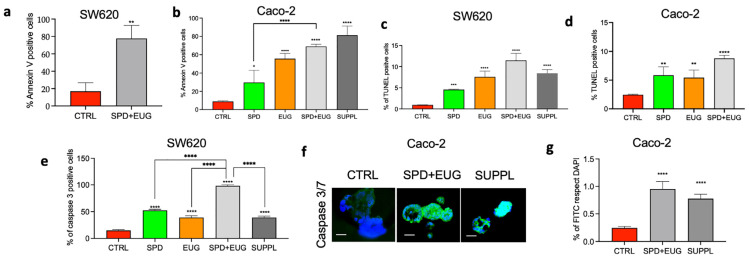
Annexin V-positive cells (**a**,**b**), TUNEL-positive cells (**c**,**d**), and caspase-3-positive cells (**e**–**g**) in 3D homotypic SW620 (**a**,**c**,**e**) and Caco-2 (**b**,**d**,**f**,**g**) spheroids following 96 h-exposure to SPD and EUG treatments as follows: SPD (150 µM), EUG (100 µM), SPD+EUG (150 µM + 100 µM) and the SUPPL (0.6 µM SPD + 50 µM EUG). All treatments were compared to the untreated CTRL. Micrograph of fluorescent green (**f**) stained caspase-3 cells in Caco-2 spheroids at ×60 mag. The nuclear material is stained blue with DAPI (4′,6-diamidino-2-phenylindole). The scale bar is 10 µm. Significant differences between treatments were reported as follows: * *p* < 0.05, ** *p* < 0.01, *** *p* < 0.001, **** *p* < 0.0001.

The contribution of apoptosis to the loss of viability was examined next. SW620 and Caco-2 spheroids were used to examine the apoptosis induction by EUG, reported only in the 2D CRCs [21,25,26,27]. The spheroids were also used to investigate the effect of SPD supplementation alone and in combination with EUG. Apoptotic activity was measured as a function of annexin-V-(FITC)-positive cells (mitochondrial membrane disruption), TUNEL-positive cells (DNA fragmentation), and caspase-3-positive cells (a major inducer of proteolytic degradation), respectively. All measurements were made after a 96 h-exposure to the treatments. SPD, EUG, SPD+EUG, and the SUPPL significantly increased the aforementioned apoptotic parameters compared to the untreated CTRL (Figure 3a–g).

Overall, both combination treatments (SPD+EUG, SUPPL) resulted in an 80% presence of Annexin V-positive cells in both SW620 and Caco-2 spheroids in comparison to the untreated CTRL (Figure 3a,b). Interestingly, the EUG alone was more effective than SPD (compare Figure 1g, Figure 2a and Figure 3b). SPD+EUG also revealed similar TUNEL-positive cells for both SW620 and Caco-2 spheroids (Figure 3c,d). SPD+EUG was shown to exceed the individual SPD and EUG contributions (Figure 3c,d). A 100% presence of caspase-3-positive cells in SW620 spheroids (Figure 3e) after exposure to SPD+EUG was found. Exposure to the SUPPL resulted in a significant decline in caspase-3-positive cells compared to SPD+EUG, with the number of caspase-3-positive cells being equivalent to that for EUG alone (Figure 3e). Figure 3f illustrated the fluorescent green puncta labelling of caspase 3 in Caco-2 spheroids, which was equivalent in SPD+EUG and the SUPPL (Figure 3g).

### 2.4. Effects of SPD and EUG Alone and in Combination on the Migration of SW620 and Caco-2 Cells in 2D Compared to the Untreated CTRLs

**Figure 4:**
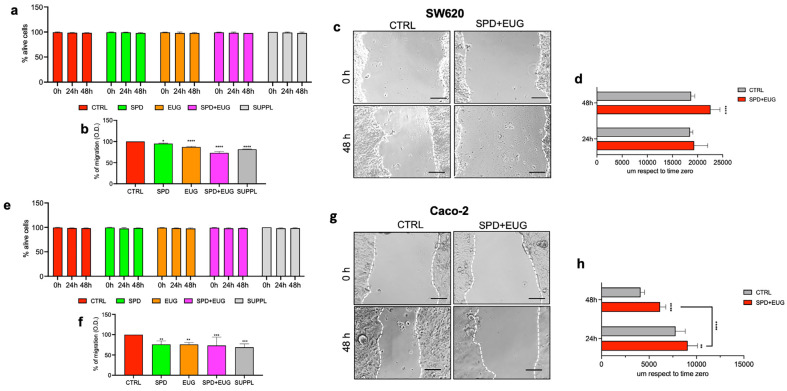
The percentage of live cells as measured by Trypan Blue at time 0 h, 24 h and 48 h for SW620 (**a**) and Caco-2 (**e**) cells and the migration percentage after 48 h in SW620 (**b**) and Caco-2 (**f**) cells in 2D following exposure to SPD and EUG treatments as follows: SPD (150 µM), EUG (100 µM), SPD+EUG (150 µM + 100 µM) and the SUPPL (0.6 µM SPD + 50 µM EUG). All treatments were compared to the untreated CTRL. Micrograph scratch diameters for the CTRL and SPD+EUG treatment in SW620 (**c**) and Caco-2 (**g**) cells at time 0 h and after 48 h at ×20 magnification. The scale bar is 100 µm. Quantification of scratch diameters for SW620 (**d**) and Caco-2 (**h**) cells after 24 and 48 h. Significant differences between treatments were reported as follows: * *p* < 0.05, ** *p* < 0.01, *** *p* < 0.001, **** *p* < 0.0001.

Cancer cells can disseminate and migrate via several mechanisms. Hence, both cancer cell migration and subsequent invasion into other tissues are integral components of metastatic disease. For this reason, we aimed to investigate the effect of the treatments on the migration percentage of SW620 and Caco-2 cells in 2D. Firstly, it was important to investigate the effect of the treatments on the vitality (Trypan Blue assay) of the SW620 and Caco-2 cells in 2D over the 48 h-exposure period. Vitality percentages of both cell lines treated with SPD, EUG, SPD+EUG, and the SUPPL were not significantly different from the untreated CTRLs at either time zero or after 24 and 48 h, respectively (Figure 4a,e). These results showed that the treatments did not interfere with the vitality of the cells, and thereby influence the migration results obtained. For the SW620 cells, all treatments significantly reduced the migration percentage compared to the untreated CTRLs (Figure 4b). EUG alone was more effective than SPD in reducing the migration percentage. For the Caco-2 cells, all treatments significantly reduced the migration percentage in comparison to the untreated CTRL (Figure 4f). The combination treatments decreased migration to a greater extent than the individual treatments (Figure 4f).

Migration of SW620 (Figure 4c) and Caco-2 (Figure 4g) cells was also examined in the SPD+EUG treatment and untreated CTRL using the scratch assay. Migration was investigated by comparing the width of the scratch, with a wider diameter being indicative of a lower migration efficiency. The scratch diameter of the untreated CTRL and SPD+EUG treatment was visualized at time zero and at 48 h in both cell types (Figure 4c,g). The CTRL cells showed a narrower scratch diameter after 48 h compared to that at time zero. This indicated an effective migration of the untreated CTRL cells to cover the scratch area (Figure 4c,g). In contrast, the scratch diameters of the two cell lines exposed to SPD+EUG for 48 h were comparable to the scratch diameters at time zero (Figure 4c,g). For SW620 cells, there was no difference in the distance of the scratch diameter between the CTRL- and SPD+EUG-treated cells after 24 h. Between 24 and 48 h, the CTRL cells did not migrate further into the scratch (Figure 4d). Interestingly, the scratch diameter of the SPD+EUG-treated cells was greater than the CTRL. This suggested that the cells pulled back away from the scratch. In contrast to the SW620 cells, the SPD+EUG-treated cells migrated less compared to the CTRL Caco-2 cells after 24 h (Figure 4d). Migration of the CTRL cells and the SPD+EUG-treated cells within the scratch proceeded between 24 and 48 h. However, migration of the SPD+EUG-treated cells was significantly less than that of the CTRL. Compared to SW620 cells, the scratch diameter was significantly lower for the Caco-2 cells, suggesting a higher migration capacity of the latter (Figure 4d,h).

### 2.5. Analysis of the Contribution Plus Interaction of SPD and EUG

Administration of SPD alone (Figure 1, Figure 2, Figure 3 and Figure 4) induced anti-CRC effects that were significantly higher than the CTRL in both cell types. Similarly, effects induced from the administration of EUG alone (Figure 1, Figure 2, Figure 3 and Figure 4) were significantly higher than the CTRL. Given that the concentrations of SPD and EUG alone were the same as those in SPD+EUG, it was possible to investigate significant differences between the three treatments. Moreover, it was possible to investigate the type of interaction occurring between SPD and EUG. This was performed for both SW620 and Caco-2 cell lines.

In the SW620 cell line, the anti-CRC effects of SPD+EUG were significantly higher than both of the individual contributions of SPD and EUG, respectively, for MTT (48 h), necrosis (48 h), TUNEL-positive cells, caspase-3-positive cells, and the migration percentage, respectively (Table 1). In the Caco-2 cell line, the same was evident for Annexin V- and TUNEL-positive cells. The synergistic interaction between SPD and EUG in the SPD+EUG treatment for both caspase-3 expression and reduced SW620 cell migration was also of great interest. It is noteworthy that, for both cell types, SPD was the predominant determinant of spheroid area and necrosis (96 h only), whereas EUG was the predominant determinant of MTT (Table 1). An antagonistic interaction was evident between SPD and EUG in the SPD+EUG-treated spheroids.

### 2.6. Discrimination Analysis of Cellular Response to SPD, EUG, SPD+EUG, and the SUPPL

**Figure 5:**
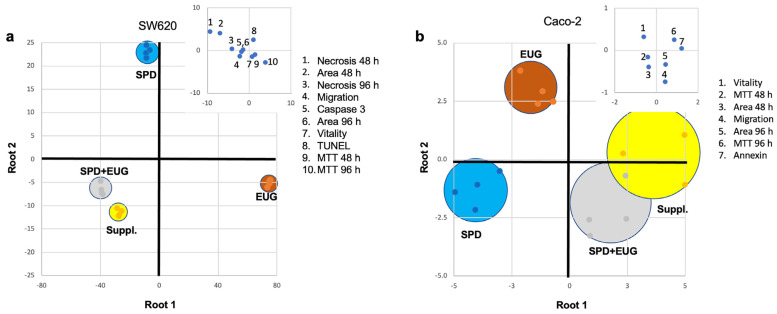
LDA analysis to examine the responses of the treatment combinations as follows: SPD (150 µM), EUG (100 µM), SPD+EUG (150 µM + 100 µM) and the SUPPL (0.6 µM SPD + 50 µM EUG) on SW620 (**a**) and Caco-2 (**b**) cell lines. The central graphs (**a**,**b**) show the positioning of the four treatments within the scatterplot in the form of circles. The dimensions of the circles reflect variability, with the smaller circles showing a lower variability and the larger circles a higher variability. The positioning of the variables (responsible for discriminating the four treatments) on the scatterplot is shown by the respective inset graphs. The positioning of the individual variables (1–10 in (**a**) and 1–7 in (**b**)) is illustrated by numbers, with each number corresponding to a specific variable which is identified from the panel in the inset graphs.

To determine which variables discriminated the four treatments the most in terms of the cellular response, a Linear Discriminant Analysis (LDA) was employed to analyze only those datasets that were complete for SPD, EUG, SPD+EUG, and the SUPPL, respectively, in both the SW620 (Figure 5a) and Caco-2 (Figure 5b) cell lines. The discriminant analysis of the four treatments was represented in two dimensions by a combined-group scatterplot. In the plot, the x-axis and y-axis showed the values of the first discriminant (Root 1) and second discriminant functions (Root 2), respectively. The cumulative percentage variance, explained for the first two roots in the discrimination of the different treatments, was equal to 98.2% for the SW620 cell line (Figure 5a). This multivariate technique showed high discrimination power, as indicated by the Wilks lambda value (0.00001), which was significant at *p* < 0.0001.

For the SW620 cell line (Figure 5a), the LDA indicated that the four treatments showed a complete discrimination (discrimination percentage of 100% at *p* > 0.05). As shown in the values of canonical functions standardized within variance, the distribution of the cases along the negative branch of Root 1 was strongly influenced by spheroid area (48 h) and necrosis (48 and 96 h). In contrast, the case distribution along the positive branch of Root 1 was mainly determined by the MTT value (48 and 96 h). The SPD+EUG and SUPPL treatments were closely scored in the bottom-left quadrant of the scatterplot (negative Root 1 and 2), with the EUG treatment positioned in the bottom-right quadrant (positive Root 1 and negative Root 2) and the SPD treatment located in the upper-left quadrant (negative Root 1 and positive Root 2), respectively.

For the Caco-2 cell line (Figure 5b), the cumulative percentage variance for the first two roots in the discrimination of the different treatments was 96.1%. The multivariate technique showed good discrimination power, as indicated by the Wilks lambda value (0.007), significant at *p* < 0.0027. As with the SW620 cell line, the discrimination percentage was 100% at *p* > 0.05. From the values of canonical functions standardized within variance, the distribution of the cases along the negative branch of Root 1 was mainly influenced by vitality, whereas the positive branch of Root 1 was strongly influenced by MTT (96 h) and migration. The treatment clustering was similar to that observed for the SW620 cell line, with the SPD+EUG and SUPPL treatments closely scored in right quadrants of the scatterplot (positive Root 1). Instead, the SPD treatment was positioned in the bottom left-quadrant (negative Root 1 and 2), with EUG in the bottom-left quadrant (negative Root 1 and positive Root 2). It is possible to observe that the Caco-2 cells exhibited a greater variability in responses, as evidenced by the major dispersion of the cases (larger circles) in the scatterplot.

### 2.7. Effects of SPD and EUG Alone and in Combination on 3D Co-Culture of Intestinal Mucosa Containing SW620/Caco-2 Spheroids Embedded in the Extracellular Matrix

**Figure 6:**
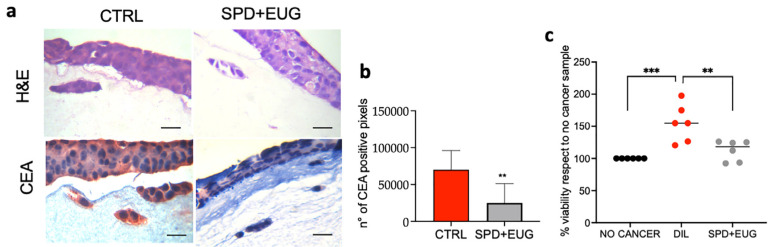
H&E staining (**a**) and carcinoembryonic antigen (CEA)-red chromogen staining quantification (**b**) of 3D intestinal mucosa models (NCM460/SW620/U937/L929 co-cultures) exposed for 24 h to SPD+EUG. All treatments were compared to the untreated CTRL. The magnification of the micrographs (**a**) was ×60. The scale bar is 10 µm. Quantification of cell viability (**c**) between NCM460/U937/L929 co-cultures (no cancer), NCM460/SW620/U937/L929 co-cultures (DIL), and NCM460/SW620/U937/L929 co-cultures treated with SPD+EUG for 24 h. The black dots indicate the positioning of the individual replicates. Significant differences were represented as follows: ** *p* < 0.01, *** *p* < 0.001.

Given that the tumor microenvironment is composed of multiple non-cancerous cells able to communicate with cancerous cells, to investigate the effects of the treatments on both the cancer spheroids and normal cells, a 3D multicellular intestinal equivalent was developed. The 3D model contained normal NCM460 enterocytes, along with monocytes (U937) and fibroblasts (L929) in the extracellular matrix (ECM). SW620 cells were seeded in the ECM, and they formed tumor masses (spheroid-like structures) over a period of 5 days, after which the SPD+EUG treatment was administered. After 24, experimental models were compared to the CTRL (containing SW620 cells and no treatment). The embedded spheroids were visualized with hematoxylin and eosin (H&E) staining (Figure 6a), and they were shown to possess the largely circular shape, as was reported before, in [16]. The presence of the carcinoembryonic antigen (CEA), a CRC-specific marker, was shown to be significantly higher in the CTRL models than in the SPD+EUG-treated models (Figure 6b). Cell proliferation using the MTT assay was analyzed on a 3D normal intestinal model without embedded spheriods, a 3D normal intestinal model containing embedded spheroids (the no treatment CTRL), and the SPD+EUG-treated model (Figure 6c). Proliferation in the healthy model was 100%, whereas inclusion of the embedded SW620 spheroids resulted in significantly increased proliferation, which was significantly reduced following exposure to SPD+EUG (Figure 6c).

**Figure 7:**
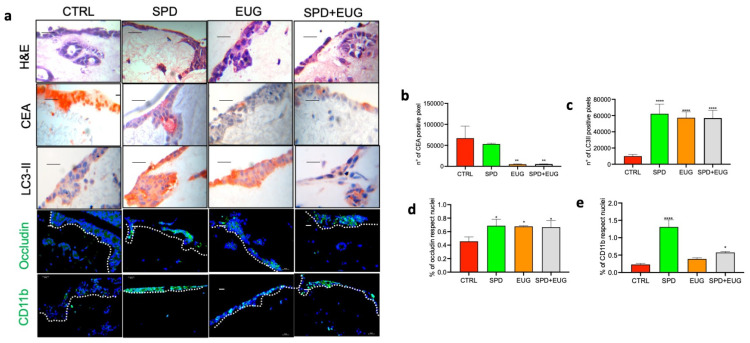
H&E staining, carcinoembryonic antigen (CEA)-red chromogen staining, LC3-II red chromogen staining, immunofluorescence green occludin and CD11b staining (**a**) of reconstructed intestinal equivalents (NCM460/Caco-2/U937/L929 co-cultures) exposed for 24 h to SPD and EUG as follows: SPD (150 µM), EUG (100 µM), SPD+EUG (150 µM + 100 µM). The magnification of the micrographs (**a**) ranged from ×40 to ×60. The scale bar is 10 µm. Quantification of CEA (**b**), LC3-II (**c**), occludin (**d**) and CD11b (**e**) expression compared to the untreated CTRL. The dashed lines indicate the division between the epithelial cells and the underlying lamina propria. Significant differences were represented as follows: * *p* < 0.05, ** *p* < 0.01, **** *p* < 0.0001.

As with SW620, Caco-2 cells were included in the reconstructed intestinal equivalents, and after 5 days the models were, similarly, treated with SPD, EUG, and SPD+EUG for 24 h. The images (Figure 7a) showed H&E staining, as well as the staining for CEA, the autophagy lipidified protein 1 light chain 3 (LC3-II) marker, the tight junction (TJ) occludin protein, and the cluster of differentiation 11b (CD11b; monocyte to macrophage transition). Results of the treatments were compared to the CTRL (containing Caco-2 cells and no treatment). The CEA staining was equivalent for both the SW620- and Caco-2-embedded spheroids in the CTRL. The SPD+EUG treatment was more effective at reducing the CEA marker in Caco-2 than in SW620 spheroids (Figure 6b and Figure 7b). EUG alone was equally as effective as SPD+EUG in reducing the CEA marker after 24 h, with SPD not showing significant differences from the CTRL. Compared to the CTRL, LC3-II staining was significantly increased in all treatments (Figure 7c). The increased LC3-II staining was representative of the entire model, which included both cancer and healthy NCM460 cells. LC3-II expression was evident in the 3D homotypic Caco-2 spheroids (Figure S1) for all treatments compared to the CTRL.

Compared to the CTRL, the overall occludin expression in the NCM640 cells was significantly increased in all treatments (Figure 7d). The immune response, resulting in the differentiation of monocytes to macrophages (presence of CD11b), was significantly higher in SPD-treated models than in the other treatments (Figure 7e). EUG did not affect the immune response, and it appeared to exert an antagonistic effect on the immune response in SPD+EUG, which was, nonetheless, significantly higher than the CTRL (Figure 7e).

## 3. Discussion

The adoption of plant-derived compounds with anti-tumorigenic efficacy is gaining interest due to the adverse effects of conventional therapies [10,11,12,13,14,15]. From in vitro and in vivo animal studies, EUG is unequivocally recognized as a potent anti-cancer compound, with a steady action over a range of mechanistic pathways that inhibit cell cycle proliferation, promote apoptotic death, and suppress metastasis, respectively [22,23,24]. For SPD, the discrepancy between the tumor growth-promoting functions of SPD (comprising the vast majority of research) versus the anti-tumorigenic functions of SPD (emerging research) led to the development of a recent hypothesis by Zimmerman et al. (2023) [31], which states that exogenous supplementation of SPD elicits anti-tumorigenic responses that override the stimulation of cancer proliferation promoted by endogenously produced polyamines (specifically SPD). Given the scarcity of research related to exogenous SPD administration, this aspect in cancer research is an important requisite [30,31], and was the focus of the present study on CRC cells cultured in 3D. In both CRC metastatic SW620 and primary Caco-2 lines, the present results verified the in vitro efficacy of EUG in the 3D spheroid form, and also showed, for the first time, the anti-tumorigenic efficacy of exogenous SPD administration alone and in combination with EUG, respectively.

Concentrations of EUG (1–100 µM) alone were shown to exert an anti-proliferative effect on homotypic SW620 spheroids. Furthermore, a role for EUG as a cell cycle inhibitor of SW620 cells cultured in 2D at higher concentrations (800 µM) was shown [21], and the anti-proliferative effect of EUG at low concentrations on cells cultured in 3D appeared to mimic that of clinically relevant PI3K inhibitors on cancer cell cultures, including SW620 [16,33,34,35]. The 100 µM EUG concentration used in the present study to induce anti-CRC activity was equivalent to the concentration used by Liu et al. [27], but significantly lower than that used previously on 2D cells [21,25,26]. Although the present study did not identify the molecular mechanisms behind the anti-CRC effect of EUG, it was shown that EUG alone (100 µM) reduced cell proliferation, viability, and spheroid area, and increased the percentage of apoptotic caspase-3/7, Annexin V-, and TUNEL-positive cells in both SW620 and Caco-2 homotypic spheroids, compared to the untreated CTRLs. These results corroborated the previous work, showing reduced viability and increased apoptosis in CRCs cultured in 2D [21,25,26,27]. Aside from the anti-growth-promoting effects, EUG alone was also shown to inhibit the metastatic-related migration percentage of SW620 cells in 2D, similarly corroborating previous reports on the anti-CRC properties of EUG [22,23,24,36].

In both cell types, SPD (150 µM) alone was shown to induce the loss of viability and reduce both the spheroid area and necrotic areas. An increase in the percentage of apoptotic caspase 3/7, Annexin V-, and TUNEL-positive cells was also evident, compared to the untreated CTRLs. Previous studies similarly showed that SPD supplementation increased apoptotic caspase-3 and Annexin markers in other cell lines [18,37]. The present results supported the hypothesis that exogenous supplementation of SPD elicits anti-tumorigenic responses [30,31]. Moreover, to the best of our knowledge, the exogenous addition of SPD was shown for the first time to exert anti-metastatic activity effects (migration percentage) on both cell types. The present results support reports on the promising anti-cancer potential of SPD [19,30,31]. Although the administration of SPD and EUG alone both exerted significant anti-CRC effects, the higher effect, exceeding that of both individual components, was observed when SPD and EUG were administered in combination.

For the first time, SPD+EUG (150 µM + 100 µM) supplementation was shown to exert significantly higher anti-CRC effects than both SPD and EUG alone. In SW620 spheroids, a synergistic effect was evident for both increased caspase-3 expression and a reduced migration percentage. Similarly, significantly improved apoptotic effects were evident for Annexin and TUNEL markers on primary Caco-2 spheroids. The molecular mechanisms behind this interaction were not investigated. It is possible that increased apoptosis was mediated via increased autophagy, which has been reported for both compounds in cancer cells [27,37]. Given that increased autophagic LC3-II expression in the Caco-2 spheroids was evident following the administration of SPD and EUG alone and in combination with each other, the role of autophagy on apoptosis warrants further attention.

SPD and EUG, when administered individually to the SW620 and Caco-2 cell lines, were shown to induce distinctive physiological/biochemical responses that differed from SPD+EUG and the SUPPL, which were similar. The anti-CRC effects of the SUPPL were largely comparable to those of SPD+EUG, even though the SPD/EUG ratios in the combination treatments ranged from 1.5 (SPD+EUG)–0.012 (SUPPL), respectively. The comparable anti-CRC effects were attributed to the absence of a dose-dependent response of SPD on cell viability in 3D. Exceptions were only evident for caspase-3 expression and the necrosis area after 96 h in SW620 spheroids. The presence of caspase-3-positive cells was significantly reduced after exposure to the SUPPL, compared to SPD+EUG. This suggested that SPD was limiting for the synergistic interaction with EUG on caspase-3 expression. Then, the darkened necrotic areas (hypoxic zones) within the SW620 spheroids were significantly higher following exposure to the SUPPL compared to SPD+EUG. This result may indicate synergistic interactions with SPD at low concentrations on alternative cell death mechanisms. It is of interest that SPD and spermine were recently shown to be potent promotors of ferroptosis [38], a recently described programmed cell death mechanism with emerging interest in cancer management [39].

In the present study, the anti-CRC effects of SPD and EUG were shown by using homotypic spheroids, which better recapitulate the 3D structure of the basic tumor compared to 2D cultures. A challenge with the use of spheroids is obtaining uniformity and reproducibility in spheroid formation, which varies according to cell lines. From the LDA analysis in the present study, Caco-2 spheroids showed a greater degree of variability in the responses to the SPD and EUG treatments, compared to the SW620 spheroids, which were more uniform. A further drawback is that homotypic spheroids do not contain the tumor microenvironment. The tumor microenvironment is multicellular, containing both stromal components (such as the ECM- and cancer-associated fibroblasts [CAFs]) and non-stromal components, and plays a vital role in the development, proliferation, and metastasis of cancer [40]. As such, an increasing number of 3D spheroid models include CAFs, endothelial cells (ECs), immune cells, and even gut bacteria, to better mimic the in vivo regulation of signaling pathways, and to gain a better understanding of cancer responses to treatments [41,42,43]. However, we considered it important to first verify the effects of SPD and EUG on homotypic spheroids. Furthermore, since anti-CRC effects by both PD and EUG were verified in the spheroids, we aimed to create a more physiologically relevant environment. For this reason, 3D reconstructed intestinal equivalents, containing embedded SW620/Caco-2 spheroids with a tumor microenvironment, were developed.

Of great relevance to the present study was that, in the reconstructed intestinal equivalents, the administration of SPD+EUG for 24 h exerted anti-CRC effects (reduced CEA marker and proliferation) on the embedded SW620/Caco-2 spheroids, whilst simultaneously exerting beneficial effects to the normal, co-cultured NCM640 enterocytes. Regarding the benefits to the surrounding cells, present results showed improved occludin expression and overall autophagy LC3-II expression compared to the untreated CTRL. Increased LC3-II expression, associated with increased autophagic flux, was demonstrated in response to both SPD and EUG in NCM640 and Caco-2 monolayers [20]. It is of note that the embedded Caco-2 spheroids also demonstrated significantly higher LC3-II expression compared to the CTRL, which may reflect autophagy-induced apoptosis; however, this point needs further clarification. Interestingly, increased autophagic activity by exogenously administered SPD was proposed to promote anti-tumorigenic effects by increasing immune cell function [31].

The present results showed that CD11b expression (reflecting monocyte to macrophage differentiation) was the highest in 3D models exposed to SPD alone. Although significantly lower than SPD alone, SPD+EUG-treated models also expressed significantly higher CD11b expression. In response to the exogenous administration of EUG and SPD, fibroblast-mediated immune effects on the embedded spheroids require more investigation. The reason for this is two-fold. Firstly, this is necessary to address the role of SPD in cancer, for which exogenous administration is reputed to impact the immune function [28,30,31]. In the present study, SPD (not EUG) stimulated the differentiation of monocytes to macrophages (presence of CD11b marker). Secondly, it is important to discern between the transient activation of fibroblasts in response to an immune response and a pathogenic response typical of CAFs [40]. Hence, the use of diagnostic markers to investigate the presence of CAFs needs to be implemented in future studies [40]. Similarly, the use of diagnostic markers to individuate cancer stem cells (CSCs) also needs to be implemented. CSCs contained within the tumor cell population are implicated behind all developmental stages of tumorigenesis, as well as recurrence. Hence, the anti-CRC efficacy of exogenous SPD and EUG administration necessitates evaluating effects on CSCs.

## 4. Materials and Methods

### 4.1. Cell Lines and Culture Conditions

The metastasis-derived SW620 cell line was purchased from the American Type Culture Collection (ATCC^®^; Manassas, VA, USA). SW620 was cultivated in high glucose Dulbecco’s Modified Eagle Medium (DMEM; GIBCO, Waltham, MA, USA) containing 10% Fetal Bovine Serum (FBS; GIBCO), 1% penicillin–streptomycin (Pen/Strep; GIBCO), and 1% L-glutamine (GIBCO). The human epithelial Caco-2 cell line (ATCC^®^ HTB-37TM), obtained from primary colorectal adenocarcinoma, was cultured with DMEM, supplemented with 10% FBS and 1% Pen/Strep. For the 3D co-cultures containing embedded SW620 and Caco-2 spheroids, a normal human colon mucosal epithelial NCM460 cell line (BeNa Culture Collection, Shanghai, China; RRID: CVCL0460); a U937 pro-monocytic, human lung myeloid leukemia cell line (ATCC^®^ CRL-1593.2); and L929 mouse fibroblasts, (ATCC^®^-CCL1) were included. Details pertaining to the culture conditions were provided previously [20]. Stock cultures of all cell lines were cultivated at 37 °C in a humidified incubator with 5% CO_2_ in tissue culture flasks (75 cm^2^; BD Biosciences, Franklin Lakes, NJ, USA), and the culture medium was changed every two days. Prior to experimentation, the cells were trypsinized, and cell density was evaluated microscopically using a Bürker counting chamber (Blaubrand, Wertheim, Germany).

### 4.2. SPD and EUG Sources

Both the SPD and EUG used in the present study were from natural sources. SPD was derived from pressed, “defatted”, dried, and milled wheat germ (a by-product of industrial wheat germ oil extraction), provided by the Targeting Gut Disease (TGD) company (Bologna, Italy). The polyamine content of the wheat germ used was determined by HPLC-MS and shown to contain SPD (750 mg/kg), putrescine (170 mg/kg), and spermine (325 mg/kg) [20]. Since SPD was the major constituent, the wheat germ component was referred to as SPD. Stock solutions of the pressed wheat germ diluted in DMEM were used for the administration of SPD alone at a concentration of 150 µM. Pure EUG (>98%), obtained from clove bud essential oil, was provided by the TGD company, and 99.5% ethanol was used to solubilize the EUG, ensuring that the selected final concentration of ethanol in the cell medium was always below 0.1%

In conjunction with the University of Bologna, the SUPPL containing both EUG and SPD was developed as part of one of the EIT FOOD projects. In brief, following the cold extraction of the oil fraction from wheat germ, the remaining pressed fibrous dry remnant (containing SPD) was used as a matrix support to absorb the EUG. SPD was measured and shown to be the predominant polyamine in the SUPPL. However, the SPD content in the SUPPL was shown to be lower than the pressed and defatted wheat germ source [20]. Information concerning the safety of the SUPPL was provided in a study by Truzzi et al. [20]. The SUPPL used in the present study contained only wheat germ matrix and EUG. No other ingredients were included.

### 4.3. Generation of Homotypic 3D Spheroids Using SW620 and Caco-2 Cell Lines and Treatment with SPD and EUG Alone and in Combination

Homotypic 3D spheroid cultures were obtained using the liquid overlay method [44]. Tissue culture (96-well) plates were coated with 100 μL 1.5% agar dissolved in DMEM. The polymerized agar was irradiated with UVB for 20 min, after which SW620 (500 cells/well) and Caco-2 cells (500 cells/well) were, respectively, seeded in 80 µL volumes. Spheroid formation was allowed to proceed for 72 h at 37 °C after plating. Firstly, a dose-dependent response of SPD and EUG on the proliferation of SW620 spheroids was performed.

Thereafter, SPD and EUG, both alone, in combination, and in SUPPL form, respectively, were diluted in DMEM. The standard concentrations administered to the SW620 and Caco-2 spheroids were as follows: 150 µM SPD alone, EUG alone, the combination treatment of 150 µM SPD + 100 µM EUG, and the SUPPL (0.6 µM SPD and 50 µM EUG). The CTRL contained only ethanol and the culture medium.

Initially, the dose-dependent response of SPD and EUG on the viability of SW620 spheroids was performed using the MTT assay after 24 h, and it was compared to that of SW620 cells (1000 cells/well) cultured in 2D. Thereafter, for both SW620 and Caco-2 spheroids, the MTT assay was performed after a 48 h- and a 96 h-exposure period to the selected treatments cited above. The spheroid areas and necrosis zones were also measured after 48 h and 96 h, respectively. Vitality and apoptosis markers were, respectively, evaluated after a 96 h-exposure to the selected treatments.

### 4.4. Construction of 3D Co-Culture Intestinal Mucosa Models (Containing Embedded SW620 and Caco-2 Spheroids) and SPD and EUG Treatments

A 3D reconstituted intestinal mucosa model, using NCM460 enterocytes with a supporting immune component of U937 monocytes and L929 fibroblasts, was constructed according to Truzzi et al. [20]. Briefly, a 24-well plate containing inserts with 0.4 µM filters (Transwell, Costar, Beijing, China) was pretreated prior to the construction of the derma in each well. A solution of DMEM was prepared to contain a count of 50,000 L929 cells, 15,000 U937 cells, and 30,000 SW620 (or 30,000 Caco-2) cells, respectively, in a volume of 50 µL for each well. The cell solution was added to a 450 µL collagen solution, and was allowed to set above the filter in each well. Each derma was then overlayed with 150,000 NCM460 cells in 50 µL. The reconstructed model was allowed to form over a 5-day period, and 500 µL fresh DMEM (10% FBS and 1% Pen/Strep) was added daily above and below the filter, respectively. On day 5, the selected treatments were administered to the upper portion of the fully formed/developed intestinal equivalent experimental models for 24 h. The comparative CTRL models included the embedded spheroids, but without SPD and EUG treatments. Additional CTRLs were generated to contain only intestinal mucosa models, but without spheroids.

After 24 h, the models were paraffin-embedded, and then sectioned according to Truzzi et al. [45], for the immunofluorescence/immunocytochemistry staining of the selected markers.

### 4.5. Viability (MTT) and Vitality (Trypan Blue) Measurements

Cell viability was measured using the MTT assay (Life Technologies, Carlsbad, CA, USA), according to the ISO 10993-5 International Standard procedure [46] in 2D, as reported previously [47]. For MTT measurements in the 3D homotypic spheroids, the method of Saltari et al. [48] was used. Instead, MTT measurements in the reconstructed 3D intestinal mucosa models were performed according to the method of Kandárová et al. [49].

Vitality of the 3D spheroids was evaluated using Trypan Blue. The spheroids were carefully resuspended in a 0.4% Trypan Blue (GIBCO) solution, and vital cells were counted using the Countess^®^II FL (ThermoFisher Scientific, Waltham, MA, USA). The results were expressed as a percentage of the untreated CTRL.

### 4.6. Determination of Spheroid Area and Necrotic Zone Areas

The anti-proliferative effect of the treatments on the SW620 and Caco-2 spheroids was evaluated by calculating the total spheroid area. In the SW620 spheroids, the pro-apoptotic/necrotic effect was measured by calculating the size area of the central necrotic zone. Total spheroid areas and necrotic zones were examined under an optical microscope (Eclipse Ts2, Nikon, Tokyo, Japan) at a magnification of ×20, and then photographed. The photographs were then examined using ImageJ2 software (Wayne Rasband, version 2.9.0/1.53t; National Institute of Mental Health, Bethesda, MD, USA), and the images were processed to pixels (300 pixels/2.54 cm). The respective spheroid areas and necrotic area zones were calculated on all of the 72 h-old spheroids (time zero), prior to exposure to each of the selected treatments for 48 h and 96 h. After each time point, the spheroid areas and necrotic zones were expressed as a percentage relative to time zero, respectively, and then expressed as a percentage of the untreated CTRL. This approach was designed to overcome any disparity attributable to differences in spheroid areas.

### 4.7. Immunofluorescence Detection of Apoptosis Markers in Homotypic Spheroids

Homotypic SW620 and Caco-2 spheroids were used for the immunofluorescence quantification of apoptotic activity. Apoptosis markers that were evaluated according to the manufacturers’ instructions included the following: translocated phospholipid phosphatidylserine (PS) with Annexin V, conjugated-to-green fluorescent PS proteins using the ANNEXIN-V Alexa Fluor 488 Invitrogen kit (ThermoFisher Scientific), DNA fragmentation using the one-step TUNEL in situ (fluorescein isothiocyanate [FITC]) apoptosis kit (Elabscience, Houston, TX, USA), and Caspase 3/7 activation using the CellEvent Caspase-3/7 Green (Alexa Fluor^®^ 488 dye) detection reagents (ThermoFisher Scientific). Visualization of positive cells was performed using a Confocal Scanning Laser Microscopy (LeicaTCS4D; Leica, Exton, PA, USA) at a magnification of ×60.

### 4.8. Cell Morphology and Immunofluorescence/Immunocytochemistry Staining of Markers in 3D Co-Culture Intestinal Mucosa Equivalents

The paraffin-embedded 3D intestinal mucosa sections (4 μm-thick) were rehydrated and stained with H&E (Bio-Optica^®^, Milan, Italy). For CEA and LC3-II (or LC3B) detection, the cells were labelled with CEA (ScyTek Laboratories, Logan, UT, USA) and LC3-II (Novus Biologicals, Centennial, MO, USA) antibodies, respectively. Immunohistochemistry was performed using fast red chromogen, according to the UltraTek Alk-Phos Anti-Polyvalent (permanent red) staining kit (ScyTek). Then, the nuclear material was stained purple with hematoxylin. The slides were examined under the microscope (MEIJI Techno Co., Ltd., San Jose, CA, USA) at a magnification of ×60 to identify CEA- and LC3-II-positive cells.

For the immunofluorescence staining, sections were labelled with occludin (Novus Biologicals) and CD11b (GeneTex Inc., Irvine, CA, USA) antibodies, respectively. Occludin and CD11b antibodies were then labelled with the Alexa Fluor 488-conjugated goat IgG secondary antibody (ThermoFisher Scientific), according to the instructions provided by the manufacturer. The images were examined using a Fluorescent Microscope (NIKON Eclipse Ni-E) at a magnification of ×40.

### 4.9. Quantification of Markers of Interest in the 3D Homotypic Spheroid and 3D Co-Culture Intestinal Mucosa Equivalents

Quantification of specific markers of interest, stained with either immunofluorescence (FITC, Alexa Fluor 488) or red chromogen, was performed by calculating the percentage of positive pixels on micrographs taken from the respective microscopes. Pictures of the cells were analyzed using ImageJ2 software (Wayne Rasband, version 2.9.0/1.53t), as described in a study by Truzzi et al. [20]. Briefly, to perform the analysis of the pixels, digital images were processed to 300 pixels/inch, and converted to 8 bits. The binary images were then further processed by the “color deconvolution” plugin to analyze the staining of the marker of interest. The selected picture was saved as a tiff for a “clean-up” procedure to eliminate artefacts with Adobe Photoshop CC (version 20.0.4). Thereafter, all fields of interest were measured with the application “Analyze particle” of ImageJ2, and the data were reported as the number of pixels. Each experiment was performed in triplicate, with three internal replicate fields analyzed for each replicate.

### 4.10. Migration of SW620 and Caco-2 Cells in Monolayer

The migration assay for all treatments was performed according to the instructions of the CytoSelect™ 24-Well Cell Migration Assay kit (Cell Biolabs, Inc., San Diego, CA, USA). The migration assay using the scratch method was also performed based on the method of Rodriguez et al. [50] for only the SPD+EUG treatment. A total of 100,000 SW620 and Caco-2 cells were, respectively, plated onto a 24-well tissue culture plate and incubated until confluence for 24 h. Cells were washed three times in Hanks’ Balanced Salt Solution (HBSS, GIBCO), and a scratch line was drawn along the monolayer with a p10 pipette tip. Plates were then washed twice with HBSS to remove all detached cells. The SPD and EUG treatments were then administered, and the plates were incubated for 24 and 48 h. Migration of SW620 and Caco-2 cells was visualized using an inverted microscope (Eclipse Ts2, Nikon) at a magnification of ×20. The results of each experiment were expressed as the mean of migrated cells from three different areas. The final results were expressed as the mean of three different experiments. 

### 4.11. Statistical Analyses

All experiments were performed in triplicate, and the data were expressed as mean values of the three different experiments. Statistical analysis was conducted using GraphPad Prism Version 10.2.3 (2024). The one-way variance (ANOVA) was used to determine any significant differences between the respective treatments and the CTRL. Using Dunnett’s multiple comparisons test, significant differences were represented as follows: ns, *p* < 0.05, ** *p* < 0.01, *** *p* < 0.001, and **** *p* < 0.0001. In the graphs, mean values expressed with stars were statistically different.

Using one-way ANOVA, significant differences between SPD alone, (150 µM), EUG alone (100 µM), and SPD+EUG (150 µM SPD+EUG 100 µM) for each of the variables studied were analyzed by one-way ANOVA and the Tukey–Kramer test at the 95% confidence level (*p* < 0.05). The interactions between SPD and EUG (indifferent, synergistic, and antagonistic) in combination were determined.

LDA, a multivariate technique that allows for the scoring of cases as a function of the first two roots, is generally used to visualize similarities and differences among objects/events. It is also a statistical method used to locate a linear combination of features (discriminant functions) that characterizes or separates two or more classes of objects or events. LDA was performed using Statistica 7.1 software (2005, StatSoft, Tulsa, OK, USA). This technique was applied to the standardized data matrix of the SW620 and Caco-2 cell line responses, respectively, for all parameters analyzed as a function of the different treatments (SPD, EUG, SPD+EUG, SUPPL). The cases (different treatments) were scored according to the first two roots (canonical discriminant functions).

## 5. Conclusions

The anti-CRC effects of EUG, described in 2D cell cultures, were verified in 3D spheroid form in the present study. The efficacy of exogenous supplementation, with SPD alone and in combination with EUG, in reducing both tumor growth and metastasis was shown for the first time in both a high-grade tumor, a metastatic CRC line (SW620), and an extensively used, low-grade, primary CRC line (Caco-2). Importantly, combining SPD and EUG to form SPD+EUG promoted a synergistic reaction in augmenting apoptotic caspase-3 expression and reducing the migration percentage in SW620 spheroids. Moreover, administration of SPD+EUG to a physiologically more relevant 3D co-culture showed a dual benefit in reducing cancer spheroid proliferation and the CEA marker, and improving TJ occludin protein expression, autophagy marker expression, and immune functions. Future studies warrant an investigation of the underlying molecular mechanisms behind SPD supplementation, alone and in combination with EUG. Reiterating the admonition in a study by Zimmerman et al. [31], SPD supplementation is not advised for cancer treatment at this point in time. Future experimentation using 3D co-culture models is essential to further investigate the effects of the immune component. Moreover, the anti-CRC potential of SPD and EUG is dependent on targeting CSCs, and this aspect will also form part of the future research.

## Figures and Tables

**Table 1:** Significant differences between SPD, EUG and SPD+EUG (*p* < 0.05) and type of interaction between SPD and EUG in SPD+EUG on measured variables in SW620 and Caco-2 cells.

Variables Analyzed	SPD, EUG and SPD+EUG, *p* < 0.05	SPD+EUG ↑ Than Both SPD and EUG, *p* < 0.05	Interaction Type *p* < 0.05
**SW620**			
MTT 48 h	SPD^c^, EUG^b^, SPD+EUG^a^	Significant	Antagonistic ***
MTT 96 h	SPD^b^, EUG^a^, SPD+EUG^a^		Antagonistic *
Area 48 h	SPD^a^, EUG^b^, SPD+EUG^c^		Antagonistic ***
Area 96 h	SPD^a^, EUG^b^, SPD+EUG^b^		Antagonistic ***
Necrosis 48 h	SPD^b^, EUG^b^, SPD+EUG^a^	Significant	Antagonistic ***
Necrosis 96 h	SPD^a^, EUG^b^, SPD+EUG^a^		Antagonistic ***
Vitality	NS		Antagonistic **
TUNEL	SPD^c^, EUG^b^, SPD+EUG^a^	Significant	Indifferent ^NS^
Caspase-3	SPD^b^, EUG^c^, SPD+EUG^a^	Significant	Synergistic **
Migration %	SPD^c^, EUG^b^, SPD+EUG^a^	Significant	Synergistic ***
**Caco-2**			
MTT 96 h	SPD^b^, EUG^a^, SPD+EUG^a^		Antagonistic *
Area 48 h	SPD^a^, EUG^b^, SPD+EUG^a^		Synergistic *
Area 96 h	SPD^a^, EUG^b^, SPD+EUG^a^		Antagonistic *
Vitality	NS		Indifferent ^NS^
Annexin-V	SPD^c^, EUG^b^, SPD+EUG^a^	Significant	Antagonistic *
TUNEL	SPD^b^, EUG^b^, SPD+EUG^a^	Significant	Indifferent ^NS^
Migration %	NS		Indifferent ^NS^

The ↑ = significantly higher. Significant differences between treatments were reported as follows: NS = not significant, * *p* < 0.05, ** *p* < 0.01, *** *p* < 0.001. Statistical analysis was performed with the letters (a, b, c) representing significant differences between treatments as determined by one-way ANOVA and the Tukey–Kramer test at the 95% confidence level (*p* < 0.05).

## Data Availability

Dataset available on request from the authors.

## Supplementary Materials

The supporting information can be downloaded at: https://www.mdpi.com/article/10.3390/ijms252413362/s1.
