# Supplementary material for: Administration of Spermidine and Eugenol Demonstrates Anti-Tumorigenic Efficacy on Metastatic SW620 and Primary Caco-2 Colorectal Cancer Spheroids
Source: Int J Mol Sci. 2024 Dec 13;25(24):13362. doi: 10.3390/ijms252413362 (PMC11679521; doi:10.3390/ijms252413362)
Supplement: Supplementary file 1 [file ijms-25-13362-s001.zip › Supplementary Figure S1.pdf]

Supplementary Figure S1.

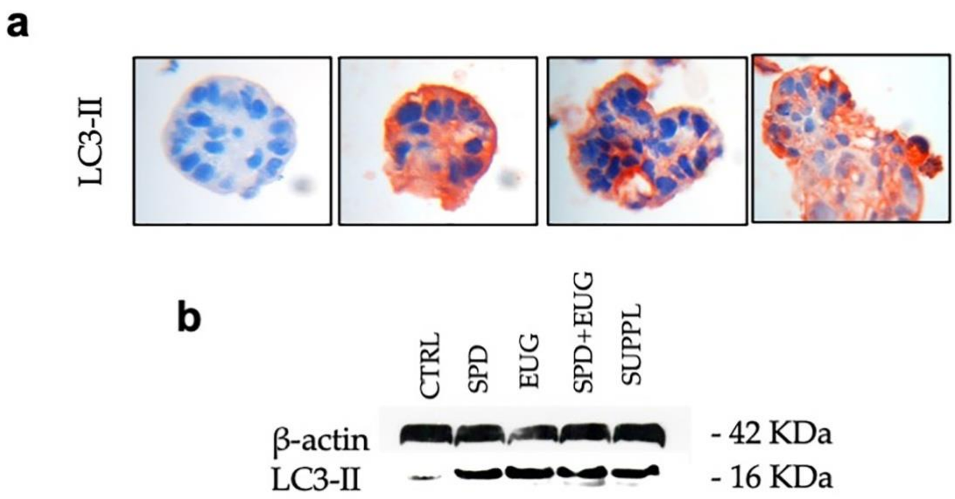

Figure S1. LC3-II expression. (a) LC3-II red chromogen staining in the spheroids of the untreated control (CTRL), spermidine (SPD), eugenol (EUG) plus SPD+EUG and (b) Western Blot LC3-II staining of the CTRL, SPD, EUG, SPD+EUG and the SUPPL after 96 h exposure to the treatments. The treatments were as follows: SPD (150 μM), EUG (100 μM), SPD+EUG (150 μM + 100 μM) and the SUPPL (0.6 μM SPD + 50 μM EUG).
